# The effect of dialysis modality on annual mortality: A prospective cohort study

**DOI:** 10.1038/s41598-024-64914-8

**Published:** 2024-06-18

**Authors:** Yae Hyun Kim, Yeonjin Kim, Nayoung Ha, Jang-Hee Cho, Yon Su Kim, Shin-Wook Kang, Nam-Ho Kim, Chul Woo Yang, Yong-Lim Kim, Jung Pyo Lee, Woojoo Lee, Hyung Jung Oh

**Affiliations:** 1 Department of Internal Medicine, Seoul National University Hospital, Seoul, Republic of Korea; 2 Department of Public Health Sciences, Graduate School of Public Health, Seoul National University, Seoul, Republic of Korea; 3 Department of Internal Medicine, Kyungpook National University Hospital, School of Medicine, Kyungpook National University, Daegu, Republic of Korea; 4 Department of Internal Medicine, Seoul National University College of Medicine, Seoul, Republic of Korea; 5 Department of Internal Medicine, Yonsei University College of Medicine, Seoul, Republic of Korea; 6 Department of Internal Medicine, Chonnam National University Medical School, Gwangju, Republic of Korea; 7 Department of Internal Medicine, College of Medicine, The Catholic University of Korea Seoul St Mary′s Hospital, Seoul, Republic of Korea; 8 Department of Internal Medicine, Seoul National University Boramae Medical Center, Seoul, Korea; 9 Department of Nephrology, Sheikh Khalifa Specialty Hospital, Ras Al Khaimah, United Arab Emirates

**Keywords:** Hemodialysis, Peritoneal dialysis, Mortality, End-stage renal disease, Nephrology, Renal replacement therapy

## Abstract

Despite numerous studies on the effect of each dialysis modality on mortality, the issue remains controversial. We investigated the hazard rate of mortality in patients with incident end-stage renal disease (ESRD) concerning initial dialysis modality (hemodialysis vs. peritoneal dialysis). Using a nationwide, multicenter, prospective cohort in South Korea, we studied 2207 patients, of which 1647 (74.6%) underwent hemodialysis. We employed the weighted Fine and Gray model over the follow-up period using inverse probability of treatment and censoring weighting. Landmark analysis was used for identifying the changing effect of dialysis modality on individuals who remained event-free at each landmark point. No significant difference in hazard rate was observed overall. However, the peritoneal dialysis group had a significantly higher hazard rate than the hemodialysis group among patients under 65 years after 4- and 5- year follow-up. A similar pattern was observed among those with diabetes mellitus. Landmark analysis also showed the higher hazard rate for peritoneal dialysis at 2 years for the education-others group and at 3 years for the married group. These findings may inform dialysis modality decisions, suggesting a preference for hemodialysis in young patients with diabetes, especially for follow-ups longer than 3 years.

## Introduction

Among the 127,068 patients with prevalent end-stage renal disease (ESRD) in Korea in 2021, 78.1% (n = 99,198), 4.4% (n = 5610), and 17.5% (n = 22,260) underwent hemodialysis (HD), peritoneal dialysis (PD), and renal transplantation, respectively. However, the prevalence of ESRD continues to increase, and the incidence rates of HD, PD, and renal transplantation were 83.6%, 5.1%, and 11.3%, respectively in 2021^1^.

Although the exact reason for the decreasing prevalence of PD in Korea is not known, this observation is not unique to Korea. The incidence of PD has decreased since the mid-1980s in the United States^2^; the preference for HD may be affected by the increasing age of patients with ESRD and insufficient information or education about the guidelines for choosing dialysis modality^3–5^.

Although the effect of each dialysis modality on survival rate has been investigated, it is still a controversial issue among nephrologists^3,4,6^. Although Korevaar et al.^7^ attempted conducting a randomized controlled trial to compare different initial dialysis modalities, the study was stopped owing to poor feasibility^7^. Less than 5% of the eligible patients agreed to be randomized. Several related studies have been conducted using diverse statistical analyses in the United States, Europe, Taiwan, and Korea^8–17^. However, the results have not been consistent.

In this study, we used data from a clinical research center (CRC) for ESRD (NCT00931970) to investigate the hazard rate of mortality in patients with incident ESRD in relation to the initial dialysis modality (HD vs. PD).

## Methods

### Study participants

**Figure 1:**
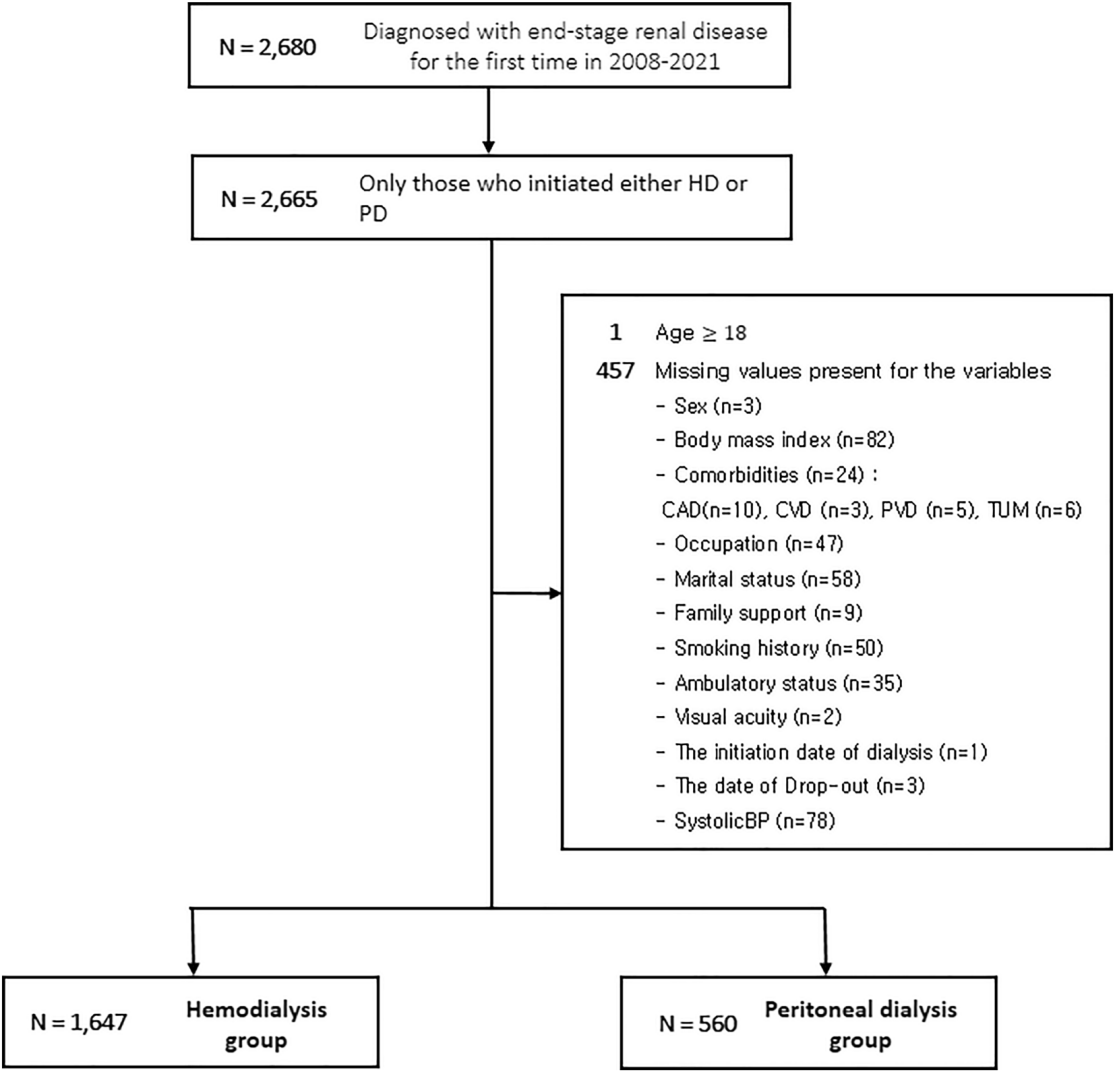
Flow diagram of the inclusion and exclusion criteria. *HD* hemodialysis, *PD* peritoneal dialysis, *TUM* tumor, *BMI* body mass index, *CAD* coronary artery disease, *CVD* cerebrovascular disease, *PVD* peripheral vascular disease, *BP* blood pressure.

The CRC for ESRD cohort was a nationwide, multicenter, prospective cohort of patients with ESRD undergoing dialysis in South Korea. The cohort began registering patients with ESRD for dialysis in July 2008; 31 hospitals in South Korea are currently participating in the study. To be eligible for inclusion in the primary cohort, patients must have been at least 18 years old at the time of enrollment. We excluded 458 patients due to missing information. The patient inclusion and exclusion processes are described in Fig. 1.

### Data collection

Data were collected from the CRC database for outcome analyses. Baseline characteristics included age, sex, body mass index (BMI), estimated glomerular filtration rate (eGFR), comorbidities, primary renal disease, occupation, marital status, family support, level of education, smoking history, ambulatory status, and visual acuity. Dialysis modality was defined as the modality used at the initiation of dialysis. Regarding the primary renal disease, five categories were investigated: diabetes mellitus (DM, category 1); hypertension (HTN, category 2); interstitial nephritis, pyelonephritis, primary glomerulonephritis, secondary glomerulonephritis, and vasculitis (category 3); cystic, hereditary and congenital disease (category 4); and miscellaneous conditions, neoplasm, tumors, and others (category 5). Comorbidities included coronary artery disease (CAD), DM, cerebrovascular disease (CVD), peripheral vascular disease (PVD), tumor, and HTN. Hospital types comprised two categories: university hospital and others. In the occupational survey, three categories were investigated: blue-collar workers, white-collar workers, and others. In addition, we collected data on family support and classified patients as thoroughly supported, 50–100% supported, < 50% supported, and independent. Considering level of education, we investigated two categories: college education or higher and others. Ambulatory state was recorded in four categories: normal, walking with assistance (e.g., a person, cane, or walker), requiring a wheelchair, and bedridden. Among the participants, 482 people had missing values of eGFR, which were imputed using MissForest^18^. Directed acyclic graphs (DAGs) were used to identify the confounders; they are shown in Supplementary Figure S1.

### Clinical outcomes

The primary outcome was time to death from any cause following dialysis initiation. Transplantation was considered a competing event. Transplantation time was determined using registration on a list of transplant recipients.

### Statistical analysis

We compared the hazard rates between the patients who underwent HD and those who underwent PD. To reduce bias arising from differences in baseline characteristics between the HD and PD groups, we employed both inverse probability of treatment weighting (IPTW) and censoring weighting (IPCW). Stabilized weights were used for IPTW where the denominator was calculated by the logistic regression model of age, sex, BMI, eGFR, comorbidities, primary renal disease, hospital types, occupation, marital status, family support, educational level, smoking, ambulation, and visual acuity as independent variables. The numerator was calculated as the proportion of each dialysis group. In regard to IPCW, we used the pooled logistic regression model of the same covariates as IPTW, additionally including types of dialysis. We used the IPTW and IPCW for weighting in the data analysis. To prevent the use of extreme weights, the weights were truncated at the 99.9th percentile of their distribution^19^. Then, we checked the balance among baseline characteristics in terms of absolute standardized mean differences (ASD) before and after the weighting. We calculated the sub-distribution hazard ratio (SHR) using the weighted Fine and Gray model^20,21^ for mortality between the two dialysis groups over the follow-up period. In addition, we employed landmark analysis to identify the changing effects of dialysis in individuals who remained event-free at each landmark point^22^. We considered five landmark points: 1, 2, 3, 4, and 5 years. Using the landmark analysis, all estimates were obtained by applying IPTW and IPCW at each landmark point.

Cumulative incidence curves were estimated non-parametrically using IPTW and IPCW. Subgroup analyses were conducted of age (less and more than 65 years old), presence of DM, educational level, and marital status. All statistical tests were evaluated using a two-tailed 95% confidence interval (CI), and statistical significance was set at *P* < 0.05. All statistical analyses were performed using R version 4.2.3.

### Ethical aspects

The study protocol complied with the Declaration of Helsinki and was approved by the Institutional Review Board of Seoul National University Hospital (H-1405-060-579). The study protocol of the CRC for ESRD was approved by the Institutional Review Board of each participating center; all patients provided written informed consent.

## Results

### Baseline characteristics

Among the 2207 patients who participated in the study, 1647 (74.6%) were in the HD group, of which there were 592 deaths (179 cardiac- and 174 infection-related deaths), 106 transplants, 8650.40 person-years of observation data, and median follow-up period of 5.70 years. In the PD group, there were 167 deaths (50 cardiac- and 40 infection-related deaths), 46 transplants, 3330.11 person-years of data, and median follow-up period of 6.57 years. A comparison of cause of deaths between the HD and PD groups is presented in Supplementary Table S1.

Table 1 presents the baseline characteristics of the patients before and after IPTW. Before IPTW, age, BMI, comorbidities, primary renal disease, occupation, marital status, educational status, ambulation, and visual acuity had ASD > 0.1 between the two groups. After IPTW, these covariates were well-balanced and had ASD < 0.1, as shown in Supplementary Figure S2^23,24^. The covariate balances in each of the subgroups by age (less than 65 years old and more than 65 years old), presence of DM, level of education, and marital status are shown in Supplementary Figure S3. The covariates were well balanced within most of the subgroups, except for the subgroups of patients more than 65 years old and those with marital status categorized as “others”.

**Table 1:** Baseline characteristics unadjusted and adjusted by IPTW.

Variables	Before IPTW		After IPTW	
	Hemodialysis	Peritoneal dialysis	Hemodialysis	Peritoneal dialysis
Age, years	57.95 ± 14.19	51.56 ± 13.00	56.21 ± 14.53	54.92 ± 13.02
Sex, n(%)				
Male	1026 (62.30%)	346 (37.70%)	1021.78 (62.08%)	355.07 (64.57%)
Female	621 (37.70%)	214 (37.70%)	624.08 (37.92%)	194.81 (35.43%)
BMI, kg/m^2^	23.12 ± 3.60	22.66 ± 3.28	22.99 ± 3.59	23.02 ± 3.26
eGFR, mL/min/1.73 ${\text{m}}^{2}$	20.88 ± 19.01	20.66 ± 32.49	20.80 ± 19.42	20.46 ± 31.48
Comorbidities, n(%)				
CAD	227 (13.78%)	57 (10.18%)	212.11 (12.89%)	74.82 (13.61%)
DM	964 (58.53%)	260 (46.43%)	911.51 (55.38%)	302.71 (55.05%)
CVD	151 (9.17%)	30 (5.36%)	136.59 (8.30%)	56.29 (10.24%)
PVD	122 (7.41%)	29 (5.18%)	112.38 (6.83%)	39.18 (7.13%)
Tumor	111 (6.74%)	17 (3.04%)	95.75 (5.82%)	27.91 (5.08%)
HTN	977 (59.32%)	243 (43.39%)	913.74 (55.52%)	313.43 (57.00%)
Primary renal disease, n(%)				
Category 1	870 (52.82%)	234 (41.79%)	820.00 (49.82%)	237.37 (49.72%)
Category 2	205 (12.45%)	94 (16.79%)	221.20 (13.44%)	71.40 (12.98%)
Category 3	238 (14.45%)	115 (20.54%)	263.71 (16.02%)	90.90 (16.53%)
Category 4	54 (3.28%)	11 (1.96%)	48.71 (2.96%)	16.57 (3.01%)
Category 5	280 (17.00%)	106 (18.93%)	292.24 (17.76%)	97.63 (17.75%)
Hospital types, n(%)				
University hospital	1455 (88.34%)	484 (86.43%)	1444.96 (87.79%)	477.25 (86.79%)
Others	192 (11.66%)	76 (13.57%)	200.90 (12.21%)	72.62 (13.21%)
Occupation, n(%)				
White-collar workers	135 (8.20%)	70 (12.50%)	153.52 (9.33%)	51.27 (9.32%)
Blue-collar workers	294 (17.85%)	158 (28.21%)	335.03 (20.36%)	110.37 (20.07%)
Others	1218 (73.95%)	332 (59.29%)	1,157.31 (70.32%)	388.23 (70.660%)
Marital status, n(%)				
Married	1189 (72.19%)	428 (76.43%)	444.04 (26.98%)	146.80 (26.70%)
Others^#^	458 (27.81%)	132 (23.57%)	1201.82 (73.02%)	403.07 (73.30%)
Family support^##^, n(%)				
Thoroughly supported	300 (18.21%)	86 (15.36%)	288.64 (17.54%)	93.66 (17.03%)
50 ~ 100%	849 (51.55%)	270 (48.21%)	838.02 (50.92%)	284.37 (51.72%)
< 50%	310 (18.82%)	123 (21.96%)	321.56 (19.54%)	106.20 (19.31%)
Never supported	188 (11.41%)	81 (14.46%)	197.63 (12.01%)	65.65 (11.94%)
Educational level, n(%)				
College graduation or higher	418 (25.38%)	197 (35.18%)	461.46 (28.04%)	159.24 (28.96%)
Others	1229 (74.62%)	363 (64.82%)	1184.40 (71.96%)	390.64 (71.04%)
Smoking, n(%)				
Never	895 (54.34%)	321 (57.32%)	905.45 (55.01%)	289.57 (52.66%)
Past smoker	569 (34.55%)	187 (33.39%)	565.14 (34.34%)	198.70 (36.14%)
Current smoker	183 (11.11%)	52 (9.29%)	175.26 (10.65%)	61.61 (11.20%)
Ambulation, n(%)				
Bed-ridden or in need of help	256 (15.54%)	43 (7.68%)	223.93 (13.61%)	73.97 (13.45%)
Self-ambulation	1391 (84.46%)	517 (92.32%)	1421.93 (86.39%)	475.91 (86.55%)
Visual acuity, n(%)				
No issues	1333 (80.94%)	477 (85.18%)	1350.48 (82.05%)	455.84 (82.90%)
Any issues	314 (19.06%)	83 (14.82%)	295.38 (17.95%)	94.03 (17.10%)

*CAD*, coronary arterial disease; *DM*, diabetes mellitus; *CVD*, cerebrovascular disease; *HTN*, hypertension; Category 1, diabetes mellitus; Category 2, hypertension; Category 3, interstitial nephritis, pyelonephritis, primary glomerulonephritis, secondary glomerulonephritis, and vasculitis; Category 4, cystic, hereditary, and congenital disease; Category 5, miscellaneous conditions, neoplasm, tumors, and others;

^#^Others; every status except for marriage.

^##^Family support.

### The impact of dialysis modality on mortality

**Figure 2:**
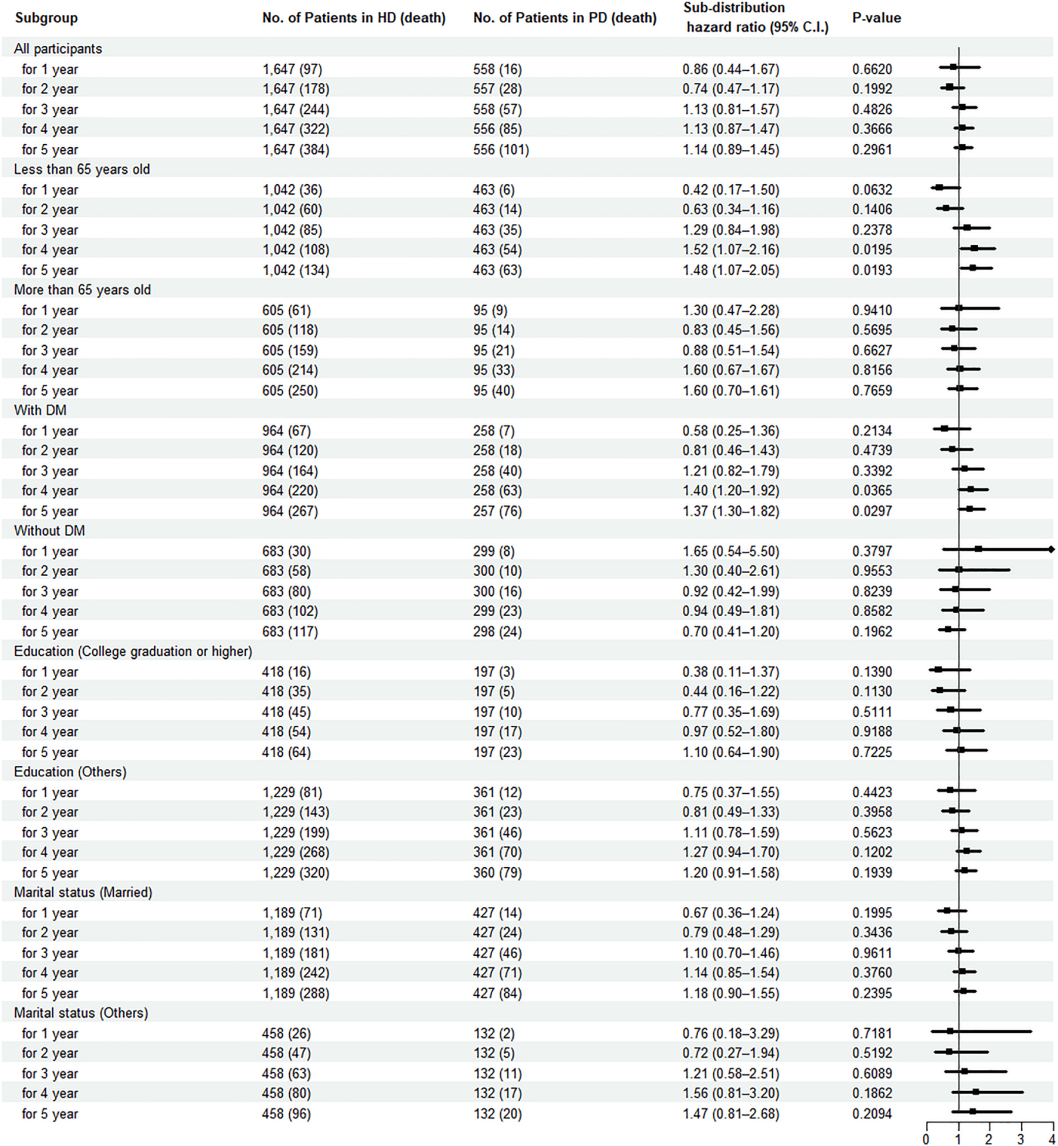
Forest plot for sub-distribution hazard ratios of mortality indicating IPTW and IPCW. *HD* hemodialysis, *PD* peritoneal dialysis.

Using the weighted Fine and Gray model of different follow-up durations, there was no significant difference in the hazard rate between the two groups (Fig. 2). However, there were significant findings using the subgroup analysis (Fig. 2). We identified a significantly higher risk in the PD group among patients less than 65 years old. Regarding 4-year follow-up, SHR was 1.52 (95%CI = 1.07–2.16, *P* = 0.0195); 5-year follow-up, SHR was 1.48 (95%CI = 1.07–2.05, *P* = 0.0193). In regard to participants with DM, the PD group had significantly higher risk of mortality than did the HD group during the 4-year (SHR [95% CI] = 1.40 [1.20–1.92], *P* = 0.0365) and 5-year follow-up (SHR [95% CI] = 1.37 [1.30–1.82], *P* = 0.0297). The analysis of the group aged more than 65 years old and without DM revealed no significant differences. In addition, there was no significant difference in the hazard rate between the two dialysis groups, irrespective of educational level and marital status. Cumulative incidence plots of all subgroups are shown in Supplementary Figure S4-S5.

### Landmark analysis for mortality according to dialysis modality

**Figure 3:**
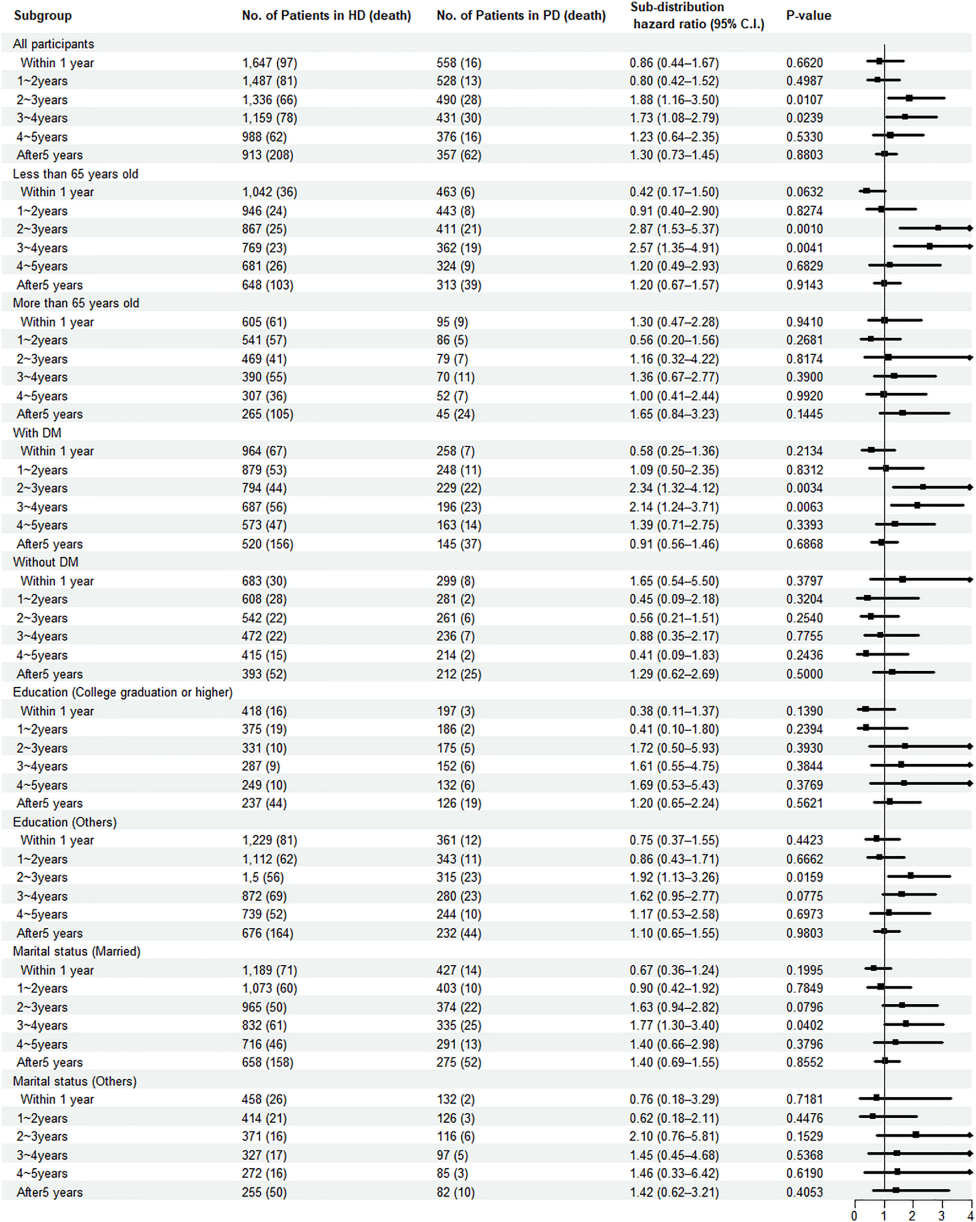
Forest plot for sub-distribution hazard ratios of the mortality showing IPTW and IPCW using the landmark analysis. *HD* hemodialysis, *PD* peritoneal dialysis, *DM* diabetes mellitus, *SHR* sub-distribution hazard ratio, *C.I.* confidence interval.

We performed a landmark analysis to investigate the effect of dialysis modality on individuals during the follow-up period. As shown in Fig. 3, the PD group had significantly higher hazard rate than did the HD group at 2 and 3 years after the first dialysis (SHR [95% CI] = 1.88 [1.16–3.50], *P* = 0.0170 and SHR [95% CI] = 1.73 [1.08–2.79], *P* = 0.0239).

Based on the subgroup analysis, the hazard rate in the PD group was significantly higher than that in the HD group at 2 and 3 years among the less than 65 years old subgroup (SHR [95% CI] = 2.87 [1.53–5.37], *P* = 0.0010 and SHR [95% CI] = 2.57 [1.35–4.91], *P* = 0.0041) and among the DM subgroup (SHR [95% CI] = 2.34 [1.32–4.12], *P* = 0.0034 and SHR [95% CI] = 2.14 [1.24–3.71], *P* = 0.0063). Moreover, the hazard rate in the PD group was significantly higher than that in the HD group at 2 years among the other education (SHR [95% CI] = 1.92 [1.13–3.26], *P* = 0.0159) and at 3 years among the married groups (SHR [95% CI] = 1.77 [1.30–3.40], *P* = 0.0402). The cumulative incidence plots estimated using landmark analysis are shown in Supplementary Figure S6-S7.

## Discussion

This study assessed the relevance of initiation of dialysis modality to hazard rate. No significant difference in hazard rate was observed between the two dialysis modalities in all participants. However, the hazard rate in the PD group was significantly higher than that in the HD group among people less than 65 years old over 4- and 5-year follow-up analysis. Moreover, we found that the hazard ratios were over 1.0 (PD vs. HD) from the 2-year follow-up period, even though statistical significance was found only after 2–3 years of follow-up. Furthermore, among the less than 65 years old group, the hazard rate in the PD group was significantly higher than that in the HD group at the follow-ups at 2–3 years and 3–4 years.

With respect to the risk of mortality, PD and HD have been considered to be equally effective as initial dialysis modalities^6,25–27^. Observational studies have also previously reported more favorable outcomes in younger patients without comorbidities who undergo PD, compared to patients who undergo HD; however, those outcomes were limited to the first 1–2 years of dialysis^28–30^, whereas HD was associated with improved survival among patients with cardiovascular disease and diabetes. Liem et al.^31^ concluded that the survival advantage of PD over HD decreased over time, with age, and in presence of diabetes as a primary disease^31^. Thus, dialysis modality conversion has remained subject to consideration; nly a few studies have investigated it^32,33^. In addition, the results should be cautiously interpreted because there were reasons for switching between modalities, such as clinical indications that precluded HD, peritonitis, and/or encapsulating peritoneal sclerosis.

Beyond considering dialysis modality conversion, we investigated the survival benefits of each dialysis modality according to the follow-up duration in the current study. To select an appropriate dialysis modality, determining the impact of dialysis modality on mortality is essential. Given that evidence from randomized controlled trials is scarce, outcome reports from cohorts or national registries are the major sources of evidence. Understanding these outcome reports requires careful interpretation due to their methodological differences, including the use of prevalent versus incident patients or Cox proportional hazards models versus landmark analysis.

In this study, we included only patients who underwent incident dialysis and reduced immortal-time bias in the patients who underwent prevalent dialysis. In addition, we used the weighted Fine and Gray regression model to reflect competing risks due to transplantation registration and performed landmark analysis to investigate the changing effects of dialysis during the follow-up period.

As previously mentioned, we did not find a significant difference in the hazard rate between the two groups during the median follow-up period of 5.9 years. However, we realized that the hazard ratios were > 1.0 when the follow-up duration was > 3 years; the hazard rate was significantly higher in the PD than in the HD group among patients less than 65 years old when the follow-up period was over 4 years. We found that more mortalities occurred in the PD group than in the HD group at 2–3 years and 3–4 years among patients in the younger age group. Although we cannot provide any exact reason for these observations, possible reasons that could have been explored might be decrease in residual renal function, recurrent episodes of peritonitis, and/or other causes of mortality. However, we could not investigate them with the limited data. Therefore, the influence of residual renal function at the time of dialysis initiation on the results could not be considered, which is a limitation of the present study. Meanwhile, consistent with several other studies, we posit that HD may be expected to offer greater survival benefit compared with PD when the follow-up period is > 2 years, particularly in younger patients.

DM is the main cause of ESRD, and several studies have demonstrated a survival advantage of HD in the presence of DM. Likewise in this study, the hazard rate was significantly higher in the PD group than in the HD group in the presence of DM. However, this only was valid in patients over a 4-year follow-up period.

In contrast, no significant differences in hazard rates were observed between the two groups when stratified by educational level and marital status, only except at 2 years. However, this needs to be interpreted cautiously because the participant’s responses were not sufficient for evaluation. The patients were asked to complete a survey. However, some were reluctant to provide answers due to privacy concerns. Thus, most patients who responded were likely to have a high level of education and family support. In the future, a more detailed survey with a well-designed study will be helpful in revealing the relationship between dialysis modality, survival benefit, educational level, and family support.

This study has several limitations. First, it was an observational study. Therefore, we had limitations in revealing a causal relationship between dialysis modality and mortality. However, we used diverse statistical analyses to address selection bias, competing risks, and non-proportional hazards models. Second, there were no guidelines for choosing the dialysis modality before enrollment. Physicians respected the preference of patients in selecting the modality. However, we educated them, showed them each case, and gave them the choice of one of the modalities except for special cases, such as patients who had contraindications for HD and PD. Furthermore, there was no consistency in dialysis initiation. We enrolled a new patient in the cohort when dialysis was initiated for ESRD. However, the decision to initiate dialysis was made by the nephrologists. The possibility of mortality could not be completely excluded because of different dialysis initiations. Moreover, the hazard rate was very low 1 year after dialysis initiation, specifically in the PD group. Thus, other factors should be considered when interpreting the survival benefit of PD within a year. However, the median follow-up period was 5.9 years, which means that the current cohort was not sufficient to reveal the long-term impact of dialysis modality on mortality. In the future, we may need to re-investigate this in a well-designed study. Finally, this study was performed only among Korean patients with ESRD, which means that the results might not be generalized: future studies involving people of diverse ethnicities will be required.

In conclusion, this study could be useful for choosing a dialysis modality in young patients, particularly those who had DM. Moreover, HD may be given preference over PD when the follow-up duration is > 3 years. However, future studies with larger populations, including those with diverse ethnicities and modality switches, are warranted.

### Supplementary Information

Supplementary Information.

## Data Availability

The datasets used and/or analysed during the current study available as de-identified participant data. Data will be shared following approval of the proposal by the corresponding author and a signed data access agreement.
