# Supplementary material for: The effect of dialysis modality on annual mortality: A prospective cohort study
Source: Sci Rep. 2024 Jun 18;14:14035. doi: 10.1038/s41598-024-64914-8 (PMC11189506; doi:10.1038/s41598-024-64914-8)

Supplementary Materials

Supplementary Table S1. Chi-square test for comparison of death between hemodialysis and peritoneal dialysis groups.

Supplementary Figure S1. Directed acyclic graph visualizing the hypothesized causal pathways.

Supplementary Figure S2. Absolute standardized mean difference before and after IPTW in all participants.

DM, diabetes mellitus; BMI, body mass index; CAD, coronary artery disease; CVD, cerebrovascular disease; PVD, peripheral vascular disease

Supplementary Figure S3. Absolute standardized mean difference unadjusted and adjusted by IPTW in the subgroups: (A) Less than 65 years old, (B) More than 65 years old, (C) With DM, (D) Without DM, (E) Highly educated (college or higher), (F) Other educational level, (G) Married, (H) Other marital status

IPTW, inverse probability treatment weighting; DM, diabetes mellitus; BMI, body mass index; CAD, coronary artery disease; CVD, cerebrovascular disease; PVD, peripheral vascular disease

Supplementary Figure S4. The cumulative incidence plots revealing IPTW and IPCW in all participants.

HD, hemodialysis; PD, peritoneal dialysis

Supplementary Figure S5. The cumulative incidence plots illustrating IPTW and IPCW in the subgroups: (A) Less than 65 years old, (B) More than 65 years old, (C) With DM, (D) Without DM, (E) Highly educated (college or higher), (F) Other educational level, (G) Married, (H) Other marital status

DM, diabetes mellitus; HD, hemodialysis; PD, peritoneal dialysis

Supplementary Figure S6. The cumulative incidence plots showing IPTW and IPCW at each landmark point in all participants.

HD, hemodialysis; PD, peritoneal dialysis

Supplementary Figure S7. The cumulative incidence plots reflecting IPTW and IPCW at each landmark point: (A) Less than 65 years old, (B) More than 65 years old, (C) DM (presence), (D) DM (absence), (E) Highly educated (college or higher), (F) Other educational level, (G) Married, (H) Other marital status

DM, diabetes mellitus; HD, hemodialysis; PD, peritoneal dialysis

Supplementary Table S1

Group	Cardiovascular Death	Infection related Death	Other cause death	$\chi^2(\mathrm{df} = 2)$	p-value
**Hemo dialysis**	179 (30.2%)	174 (29.4%)	239 (40.4%)	2.40	0.3012
**Peritoneal dialysis**	50 (29.9%)	40 (24.0%)	77 (46.1%)		

Supplementary Figure S1

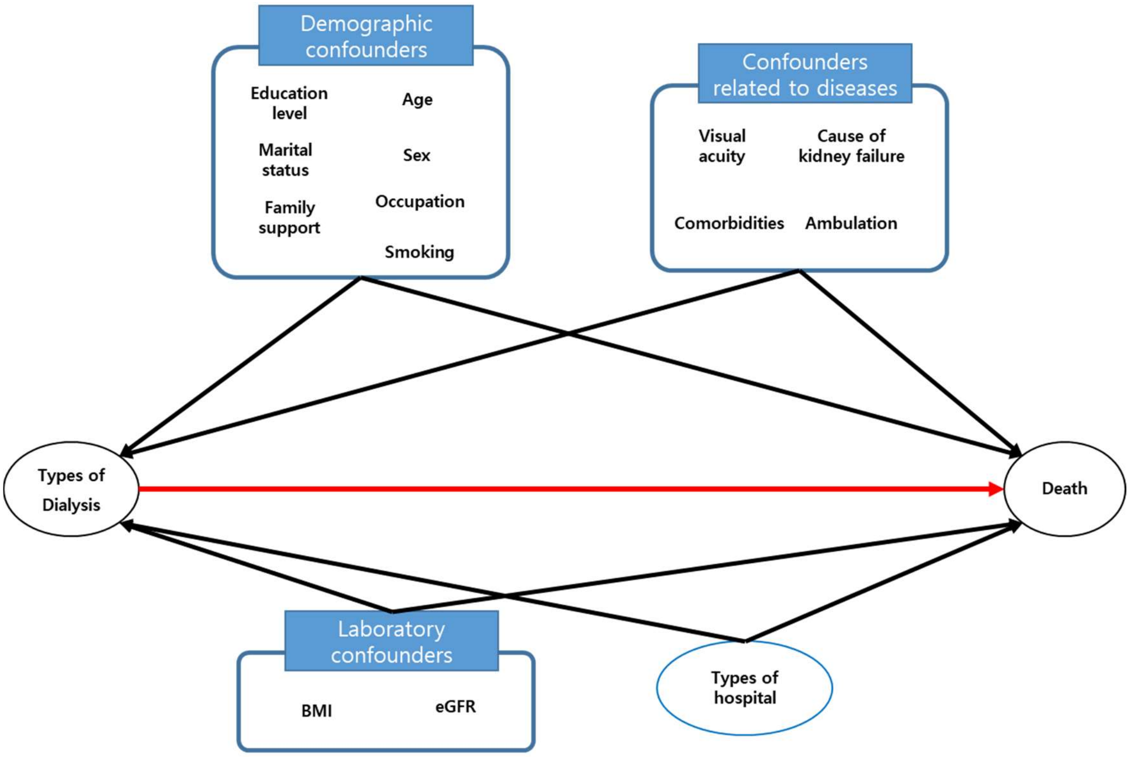

Supplementary Figure S2

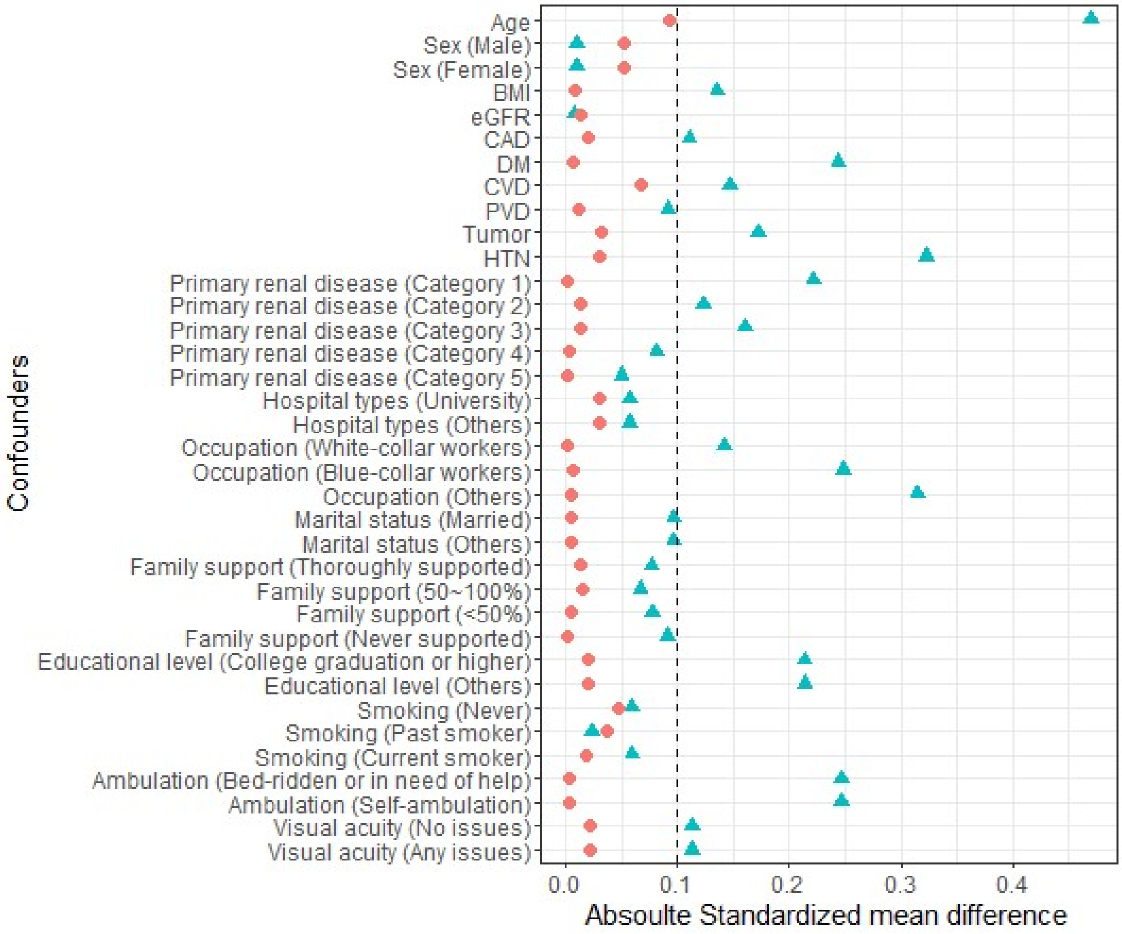

Supplementary Figure S3

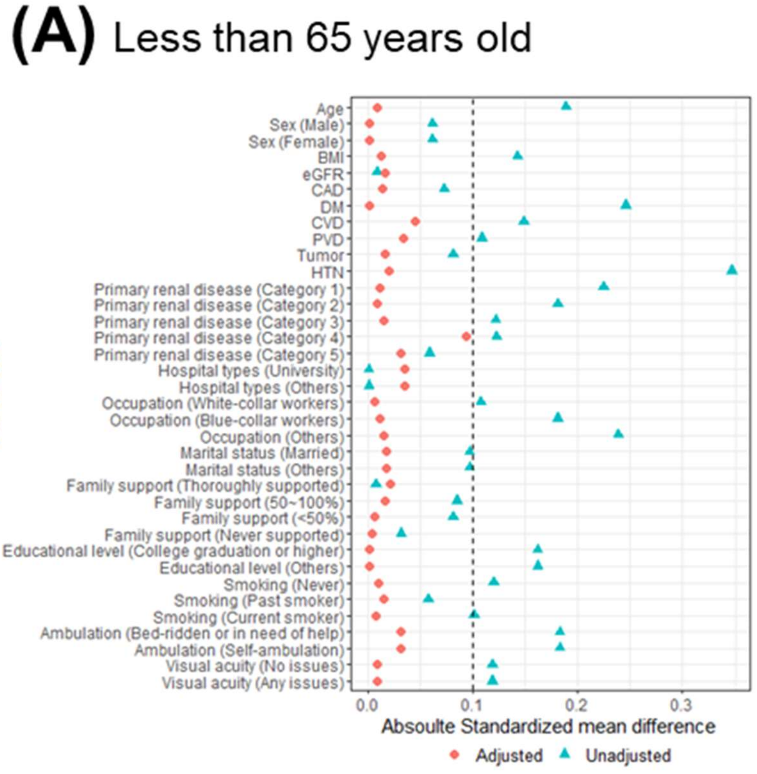

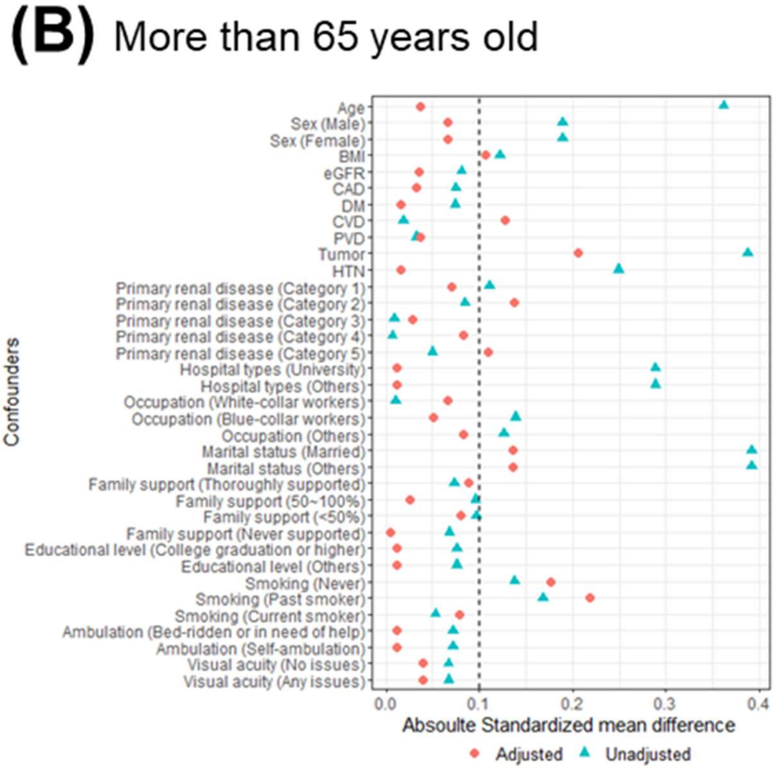

(C) With DM

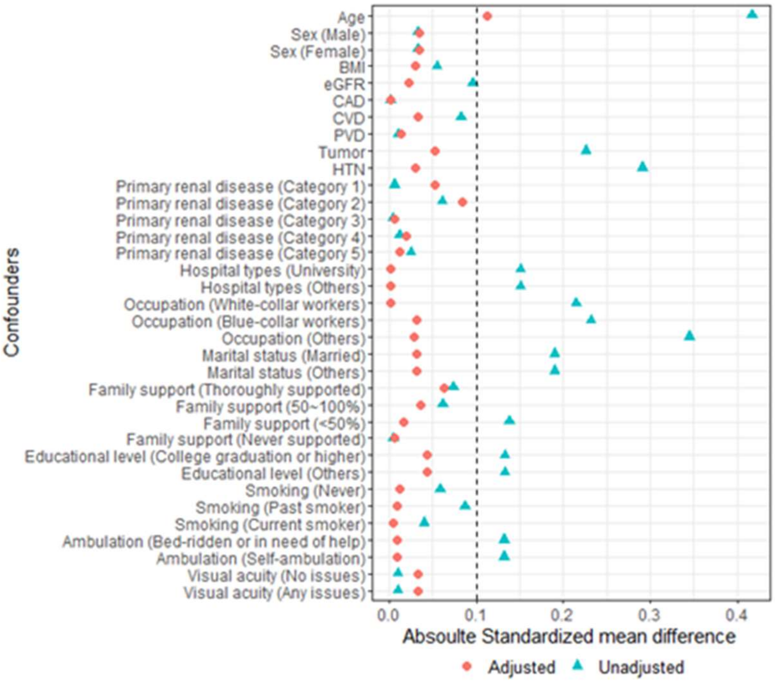

(D) Without DM

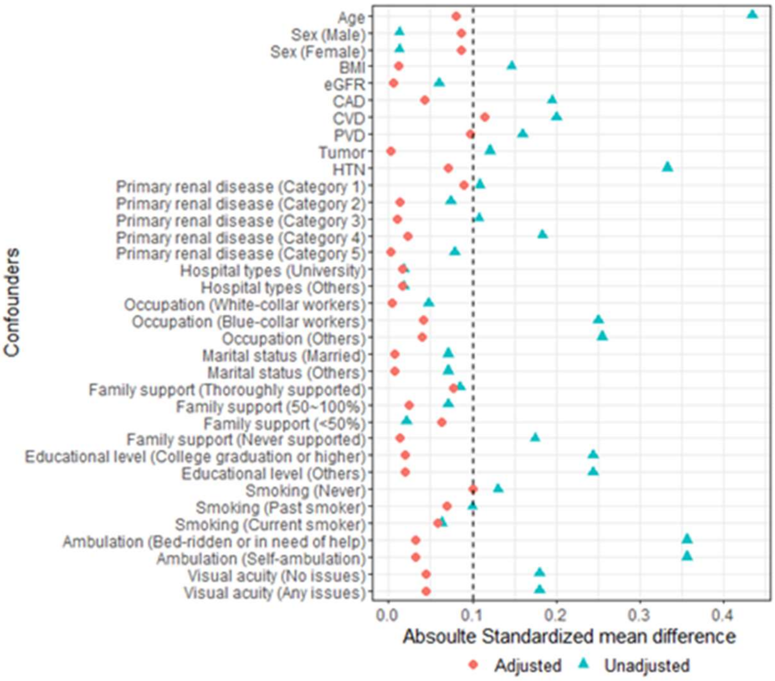

(E) Highly educated (college or higher)

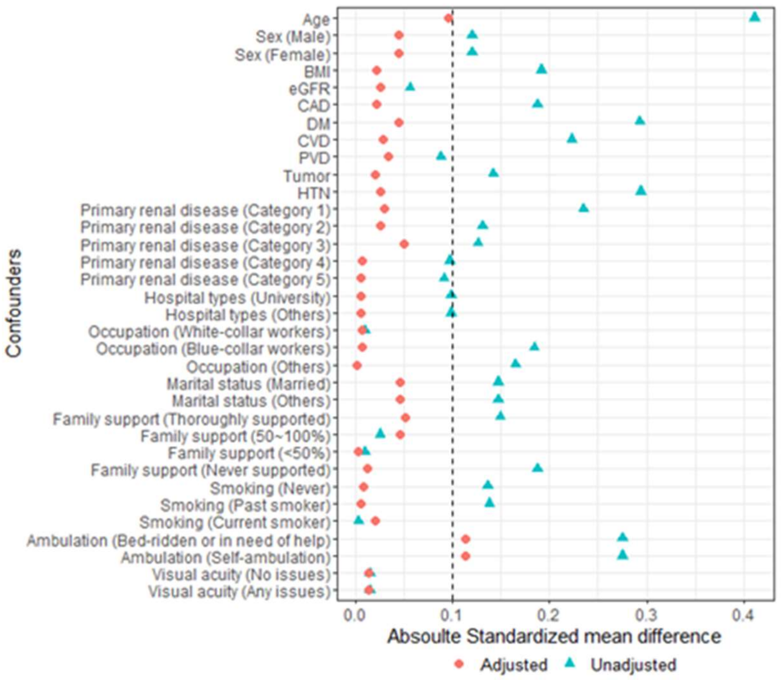

(F) Other educational level

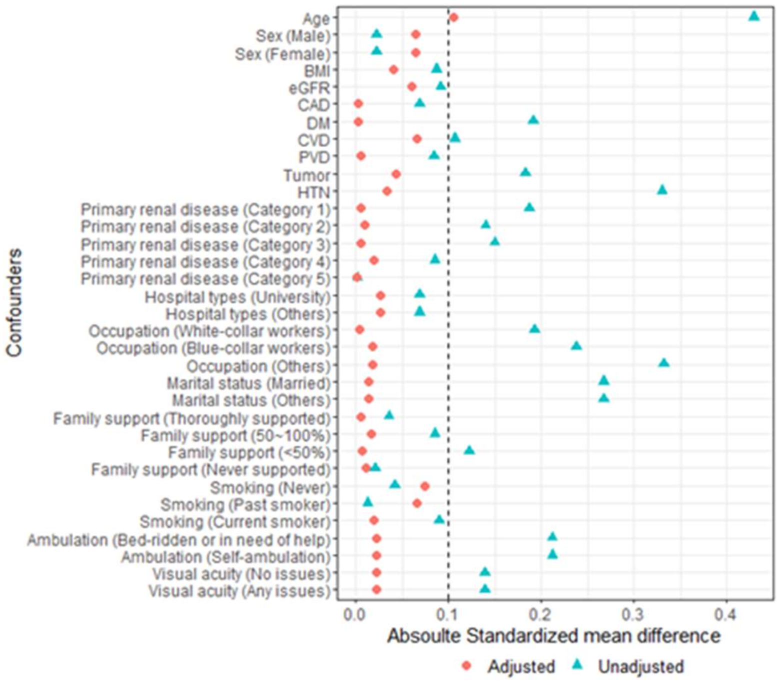

(G) Married

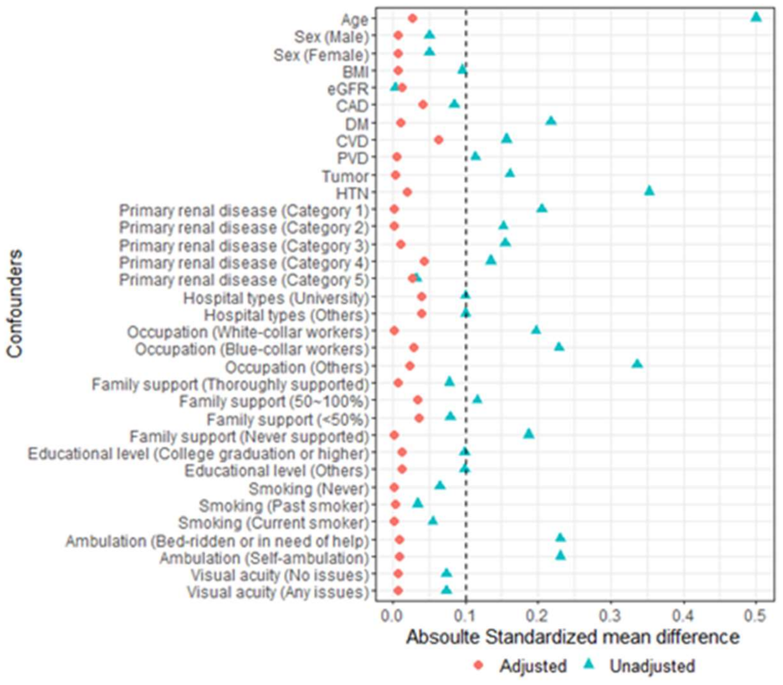

(H) Other marital status

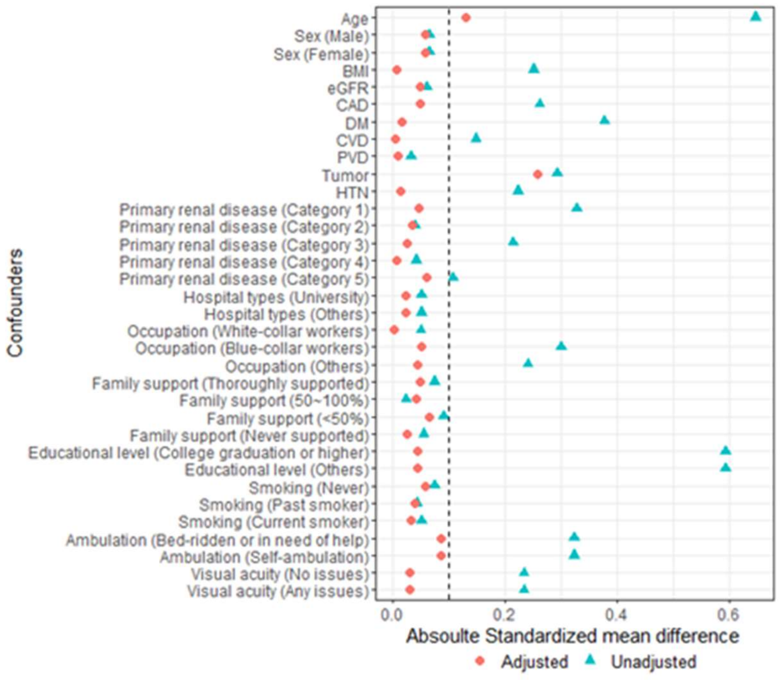

Supplementary Figure S4

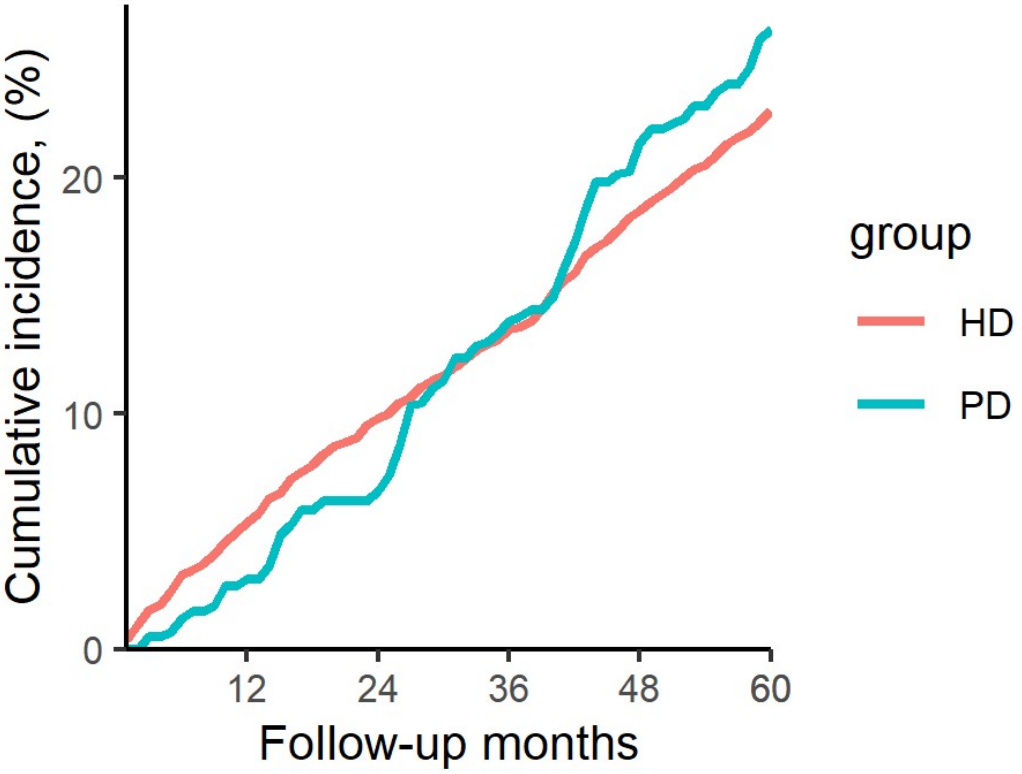

Supplementary Figure S5

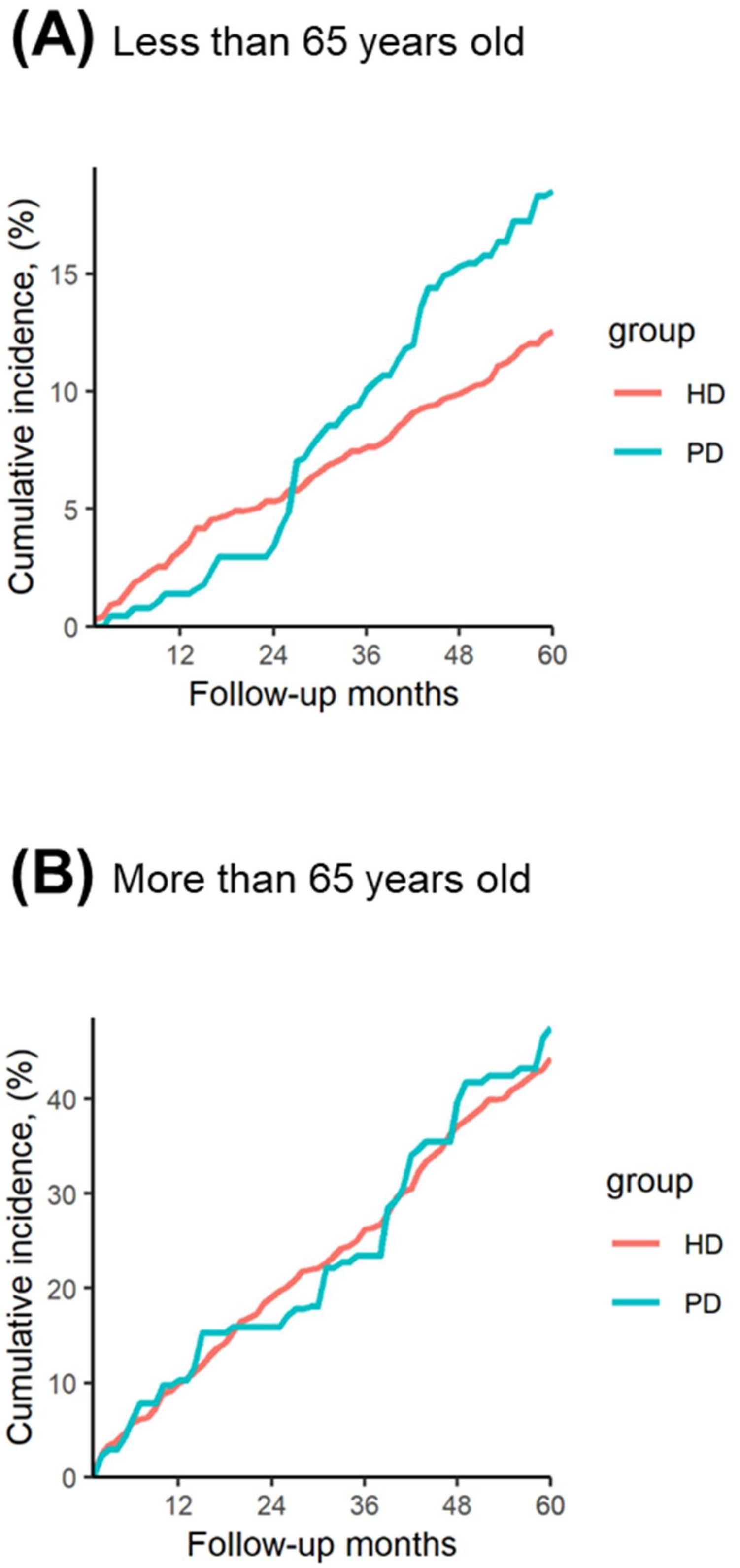

(C) With DM

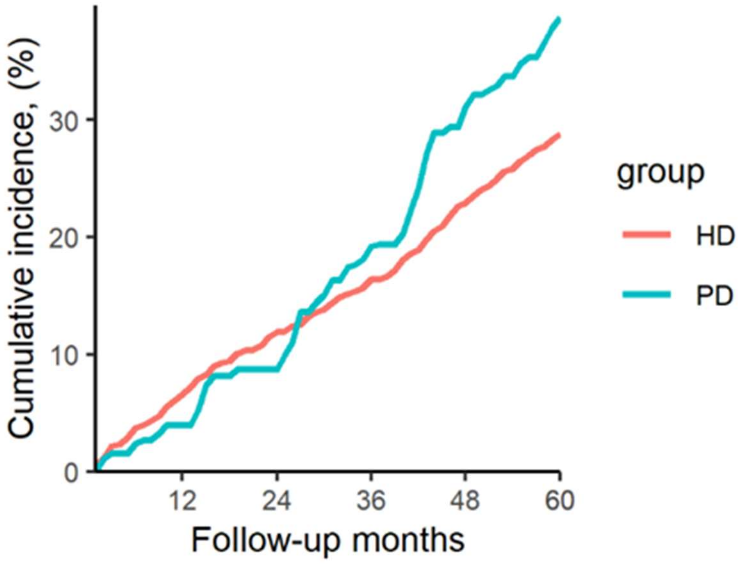

(D) Without DM

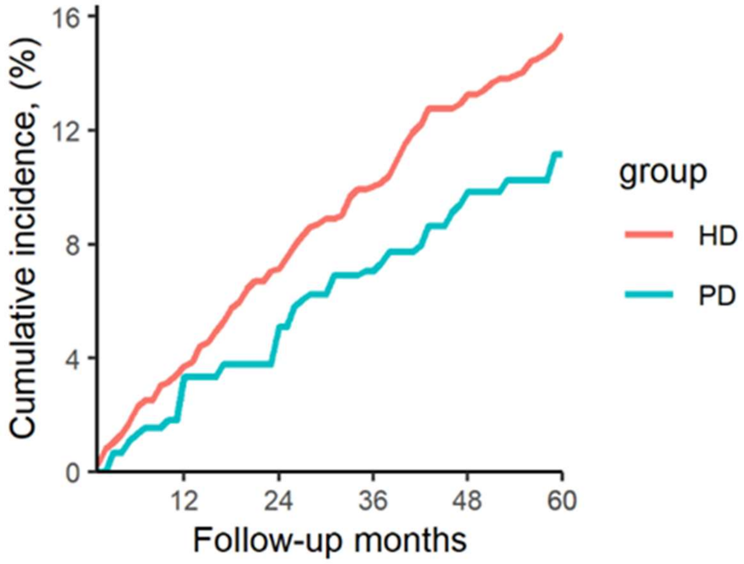

(E) Highly educated (college or higher)

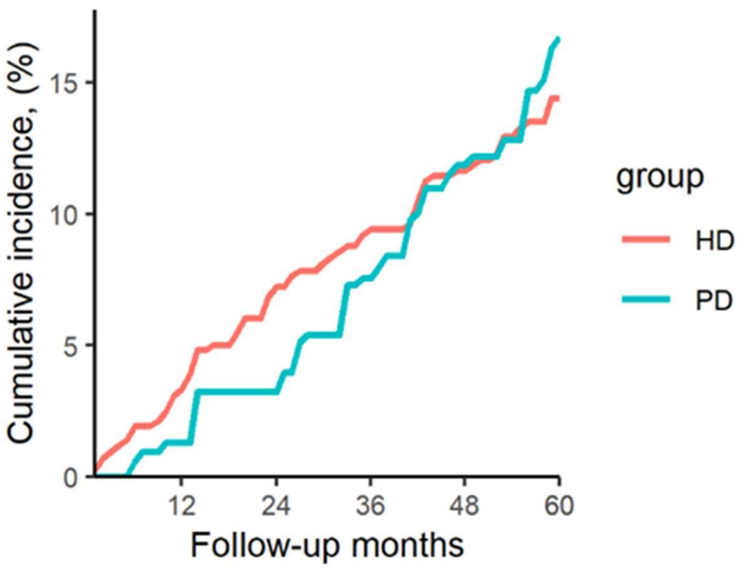

(F) Other educational level

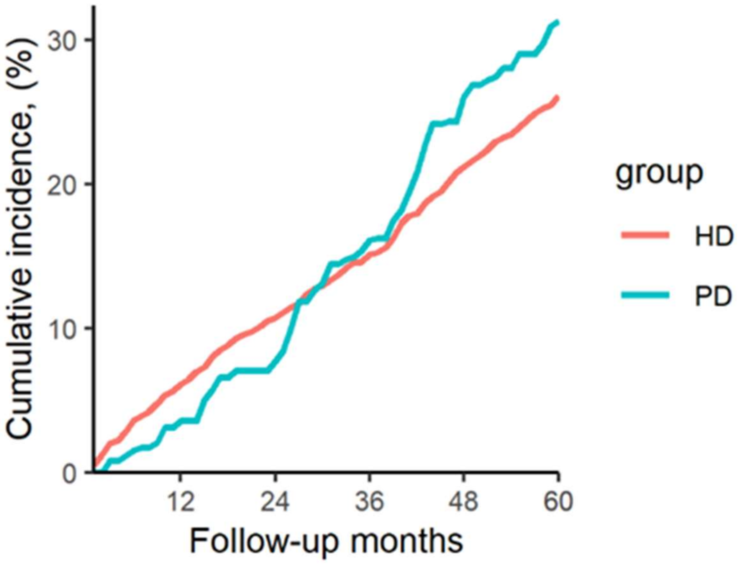

(G) Married

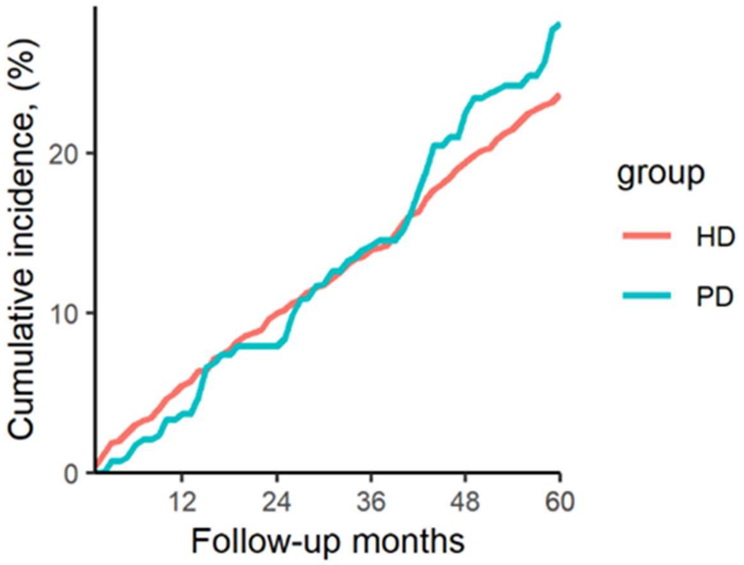

(H) Other marital status

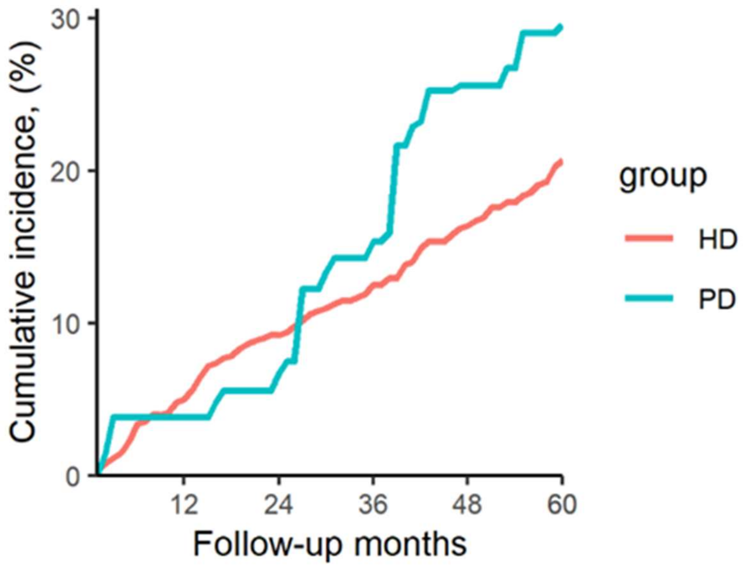

Supplementary Figure S6

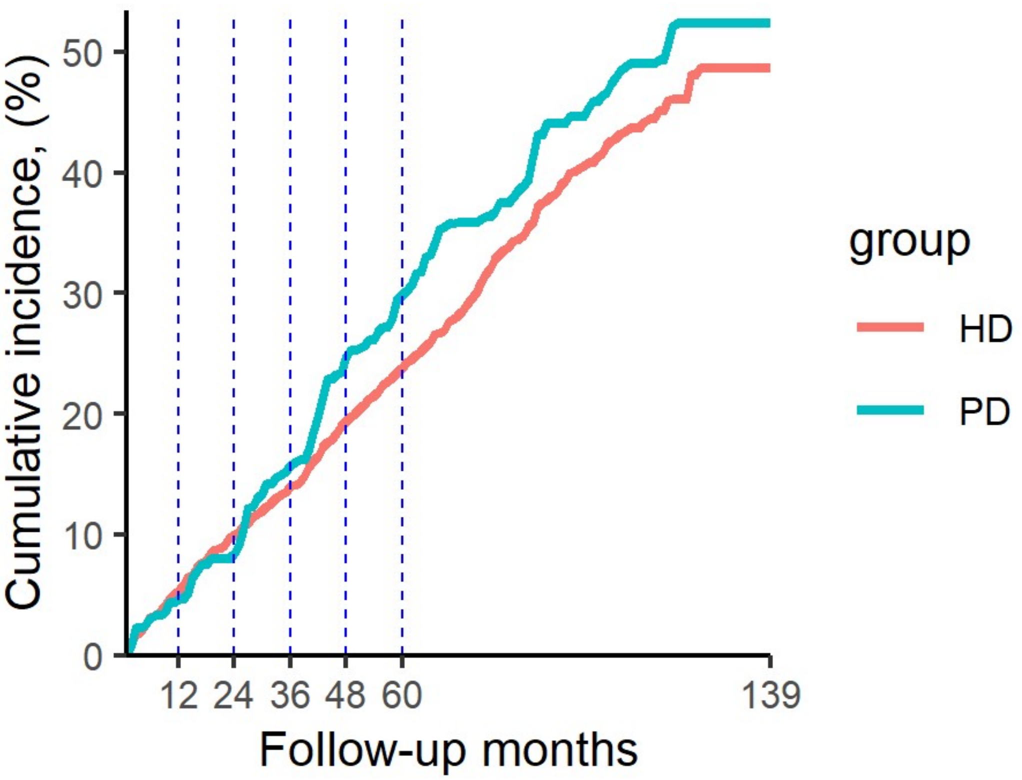

Supplementary Figure S7

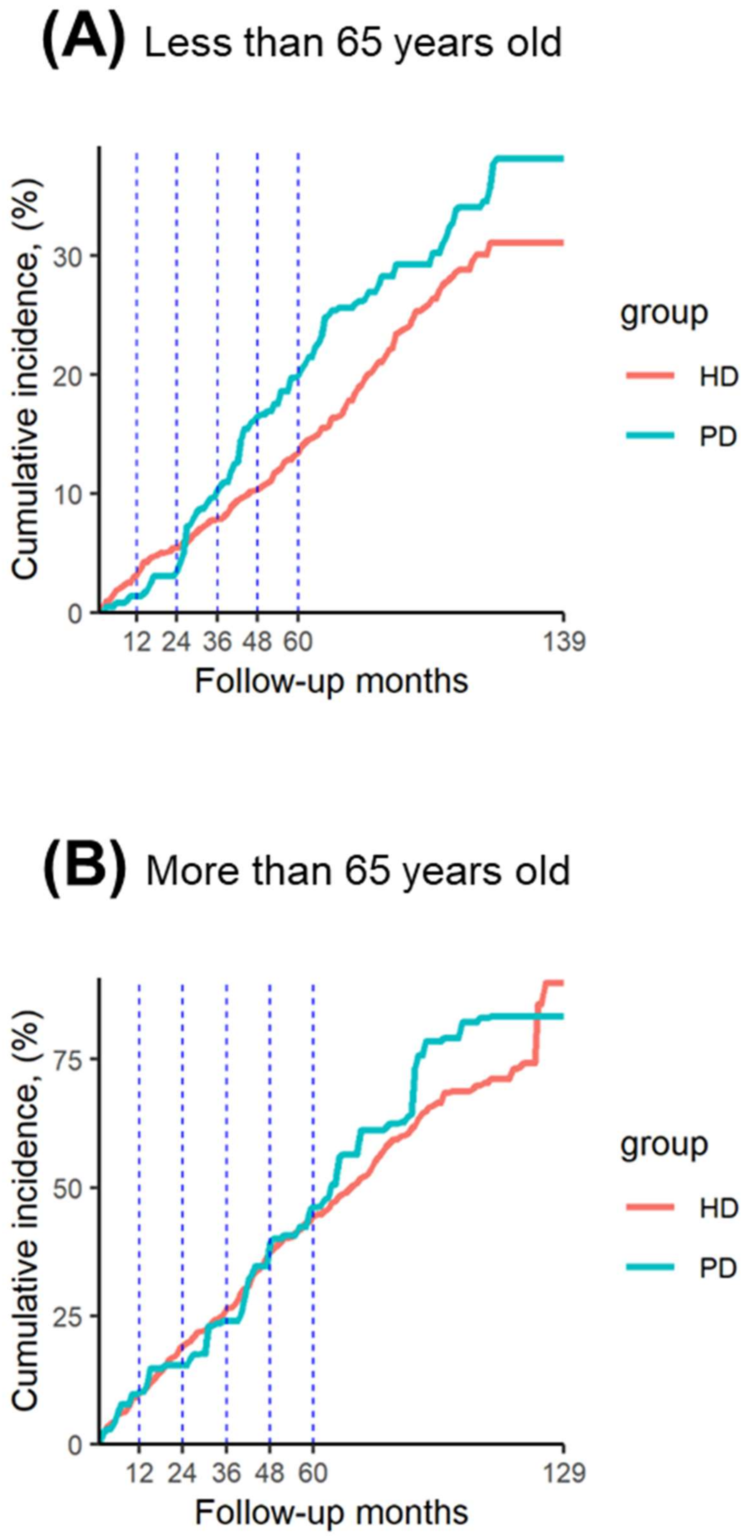

(C) DM (presence)

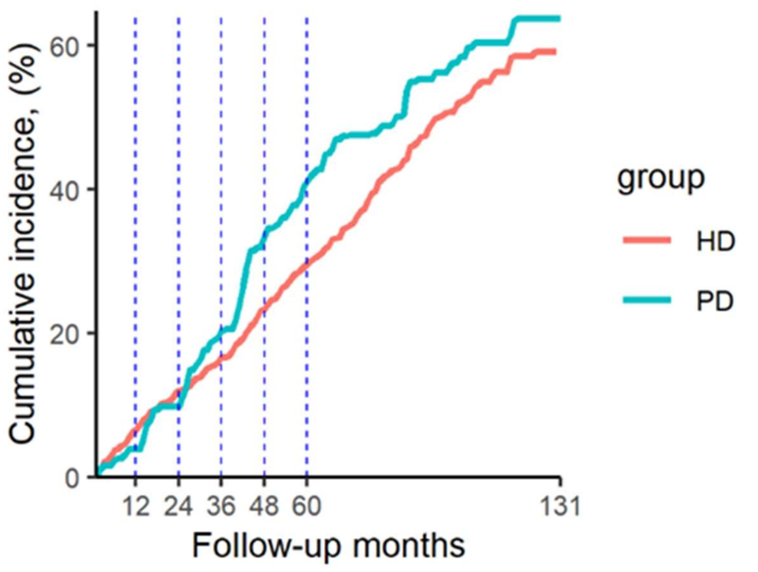

(D) DM (absence)

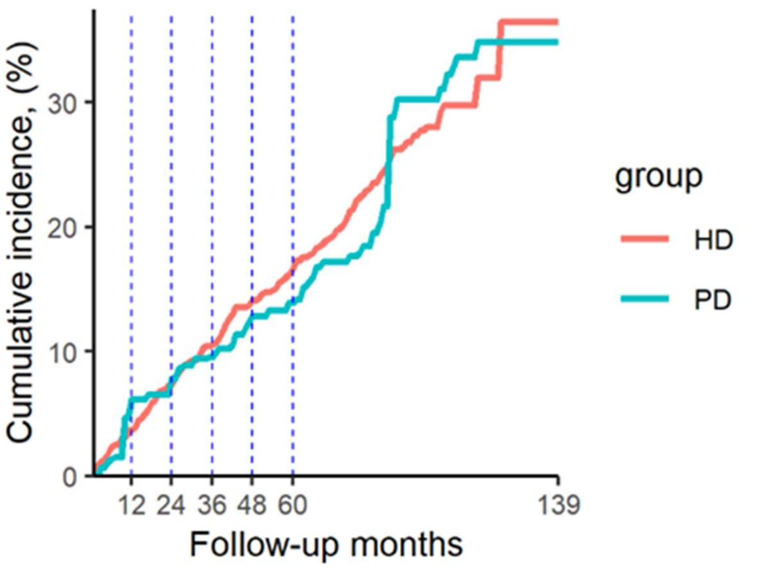

(E) Highly educated (college or higher)

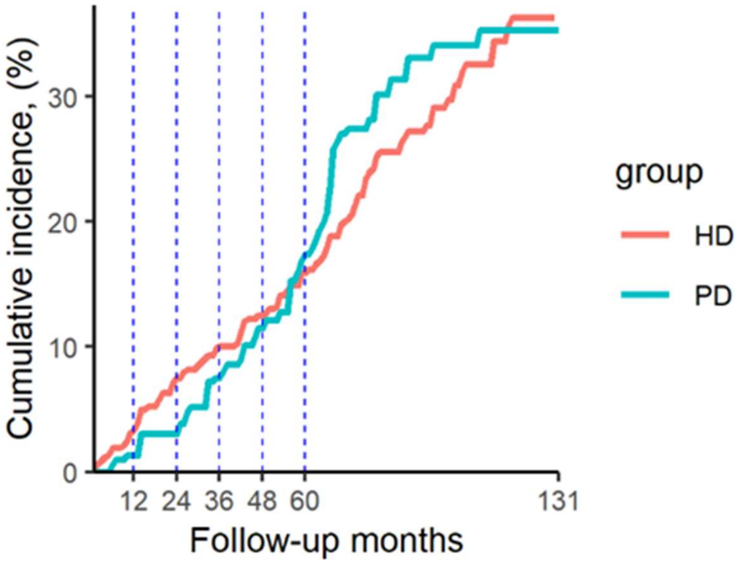

(F) Other educational level

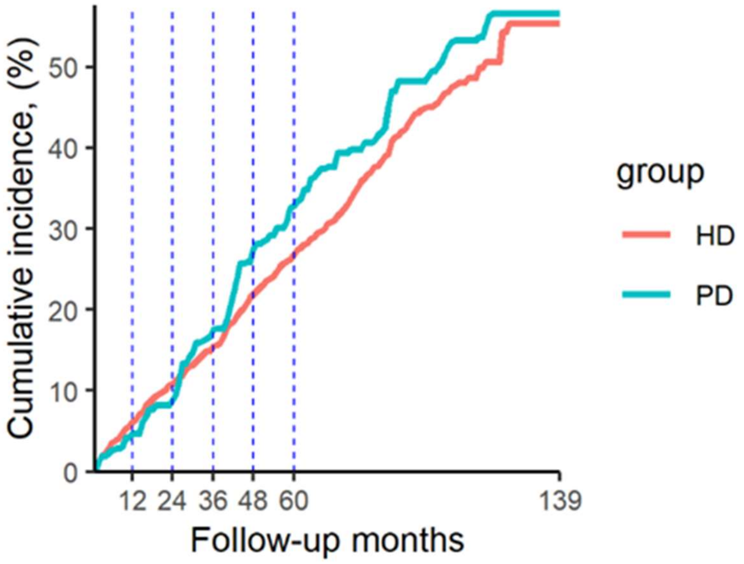

(G) Married

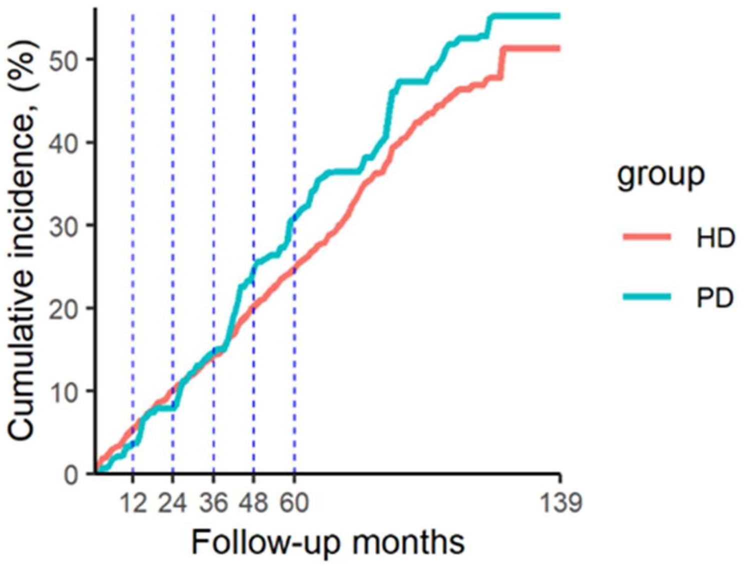

(H) Other marital status

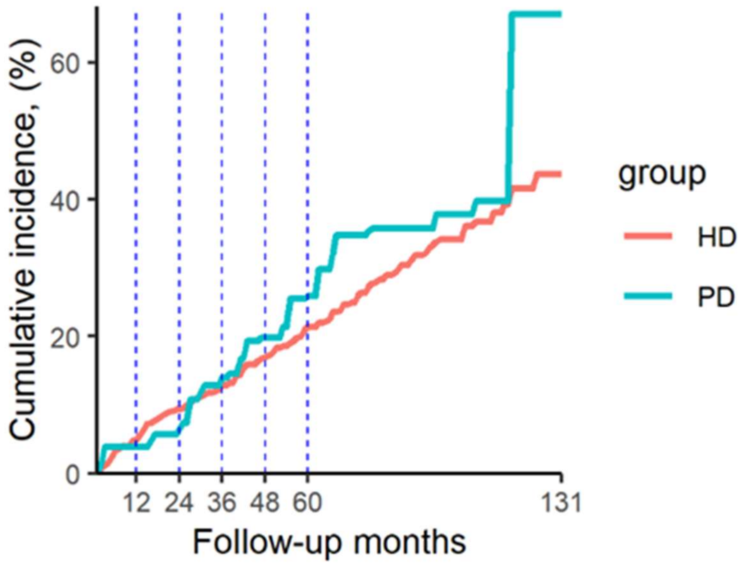

Supplement: Supplementary file 1 — Supplementary Information. [file 41598_2024_64914_MOESM1_ESM.pdf]
